# Association of Dietary Inflammatory Index with CKD progression and estimated glomerular filtration rate in the American CKD population: A cross-sectional study

**DOI:** 10.1371/journal.pone.0297916

**Published:** 2024-02-22

**Authors:** Zichen Xu, Lei Li, Luqing Jiang, Ying Zhai, Yu Tang, Daoqin Liu, Qiwen Wu

**Affiliations:** 1 Clinical Laboratory, The First Affiliated Hospital of Wannan Medical College, Wuhu, 241001, Anhui, China; 2 Department of Kidney Medicine, The First Affiliated Hospital of Wannan Medical College, Wuhu, 241001, Anhui, China; Isfahan University of Medical Sciences, ISLAMIC REPUBLIC OF IRAN

## Abstract

**Purpose:**

The number of CKD patients is on the rise worldwide, and diet has become an essential aspect influencing the treatment and prognosis of CKD. However, limited research has explored the association of the Dietary Inflammatory Index (DII) with CKD progression and the essential kidney function indicator, eGFR, in CKD patients. This study aimed to analyze the association between DII and CKD progression and eGFR in the US CKD population using data from the National Health and Nutrition Examination Survey (NHANES).

**Methods:**

This study utilized data obtained from the National Health and Nutrition Examination Survey (NHANES) spanning from 2007 to 2018, with a total sample size of 2,488 individuals. Study used multiple imputation, based on 5 replications and a chained equation approach method in the R MI procedure, to account for missing data. Weighted multiple logistic regression was used to analyze the relationship between DII and the risk of higher CKD stage and a weighted multiple regression analysis was used to assess the relationship between DII and eGFR. Weighted Generalized Additive Models and smoothed curve fitting were applied to detect potential non-linear relationships in this association.

**Results:**

In all three models, it was found that DII was positively associated with the risk of higher CKD stage (*P* < 0.0001), and an increase in DII was associated with a decrease in eGFR (*P* < 0.0001). The trend across quartiles of DII remained statistically significant, revealing a gradual elevation in higher CKD stage risk and reduction in eGFR levels for the second, third, and fourth quartiles compared to the lowest quartile (*P* for trend < 0.0001). Upon adjusting for age, gender, race, education level, poverty income ratio (PIR), marital status, body mass index (BMI), metabolic equivalent (MET) score, drinking, smoking, history of hypertension, history of diabetes, cotinine, systolic blood pressure, diastolic blood pressure, total triglycerides, and total cholesterol, we found a positive correlation between DII and the risk of higher CKD stage (OR = 1.26, 95% CI: 1.14–1.40). Further investigation revealed that an increase in DII was associated with a decrease in eGFR (β = -1.29, 95% CI: -1.75, -0.83). Smooth curves illustrated a non-linear positive correlation between DII and CKD risk, while a non-linear negative correlation was observed between DII and eGFR.

**Conclusions:**

Our study results indicate that an increase in DII is associated with an increased risk of higher CKD stage and a decrease in eGFR in all three models. In the fully adjusted model, the risk of higher CKD stage increased by 26% and the eGFR decreased by 1.29 ml/min/1.73 m^2^ for each unit increase in DII. This finding suggests that in patients with CKD in the US, improved diet and lower DII values may help slow the decline in eGFR and delay the progression of CKD.

## Introduction

Chronic kidney disease (CKD) is a heterogeneous condition characterized by structural abnormalities of the kidneys and impaired renal function [1,2]. In 2017, there were an estimated 697 million CKD cases globally, with a prevalence of approximately 9.1% in the world’s population [3]. Typically, for CKD patients, estimated glomerular filtration rate (eGFR) serves as a quantifiable indicator for assessing the decline in renal function and kidney damage [4,5]. The Kidney Disease Outcomes Quality Initiative (KDOQI) classifies CKD into five stages based on eGFR per ml/min per 1.73 m2: Stage 1 (GFR ≥ 90), Stage 2 (GFR 60–89), Stage 3 (GFR 30–59), Stage 4 (GFR 15–29), and Stage 5 (GFR < 15).

Inflammation is considered a crucial factor in the onset and progression of CKD [6]. CKD patients are prone to infections, and increased oxidative stress and elevated pro-inflammatory cytokines contribute to the establishment of a chronic pro-inflammatory state in these individuals [7]. Moreover, there is evidence suggesting that anti-inflammatory treatments can effectively reduce the risk of major adverse cardiovascular events in CKD patients [8].

Furthermore, previous research has highlighted the significance of a healthy dietary pattern in the management of CKD [9]. Consuming an anti-inflammatory diet rich in Ω-3 Polyunsaturated Fatty Acid (PUFA), vitamins and fiber has been associated with a reduced risk of proteinuria and an improvement in kidney function [10]. Conversely, the intake of nutrients generally considered pro-inflammatory, such as saturated fatty acids and sugars, is associated with an increased risk of worsening kidney function [11]. The Dietary Inflammation Index (DII) is a literature-based, population-based scoring system designed to assess the inflammatory potential of diet [12].

If the DII value is positive, the diet contains pro-inflammatory components, and if the DII value is negative, it means that the diet contains anti-inflammatory components. A higher score indicates a stronger pro-inflammatory effect, and a lower score indicates a stronger anti-inflammatory effect [13].

## Methods

### Data source and study sample

**Fig 1:**
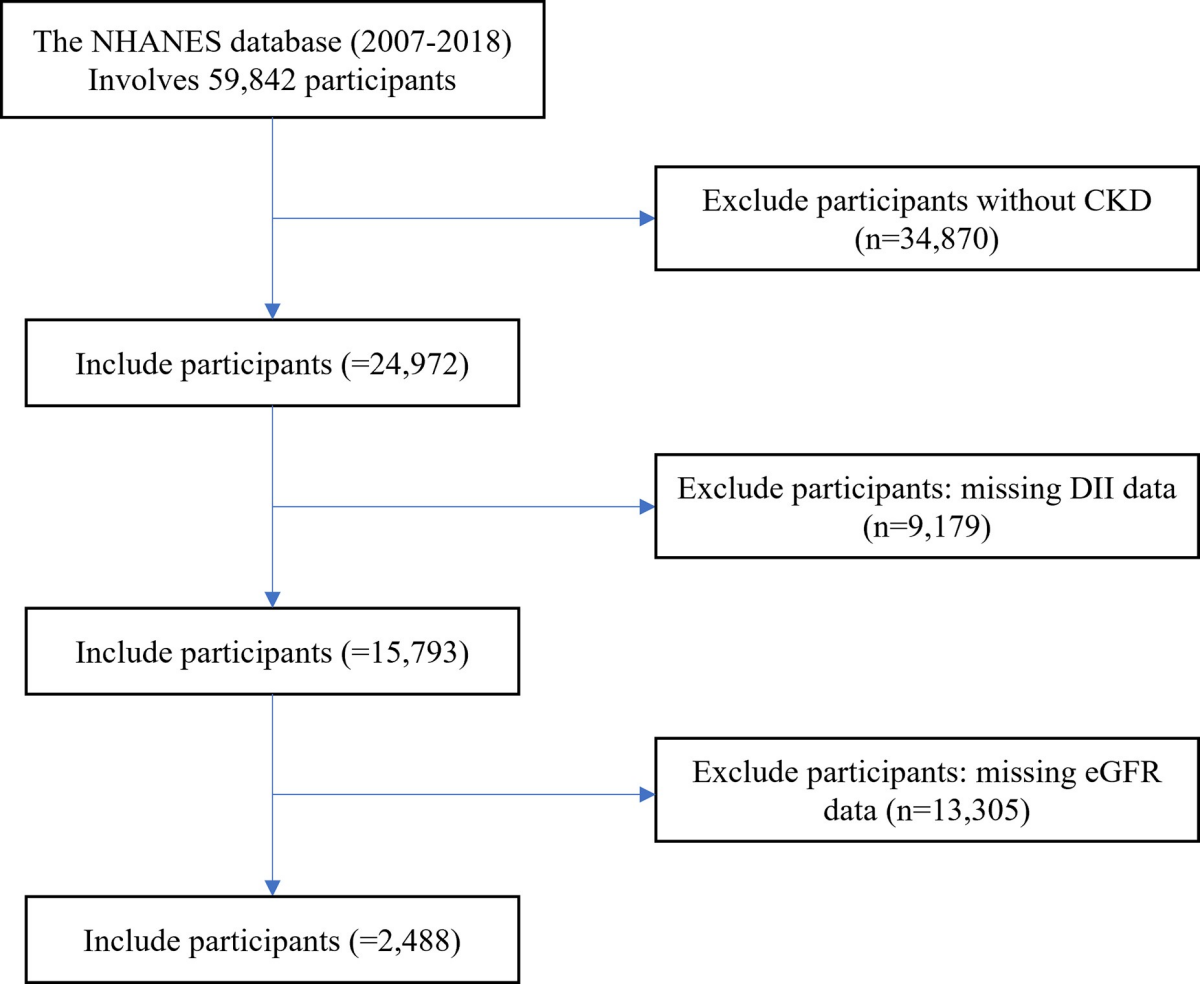
Flowchart for selecting analyzed participants. CKD, chronic kidney disease; DII, Dietary Inflammatory Index; eGFR, estimated glomerular filtration rate; NHANES, National Health and Nutrition Examination Survey.

The National Health and Nutrition Examination Survey (NHANES) is a nationwide survey conducted by the National Center for Health Statistics (NCHS) in the United States. Its primary aim is to assess the health and nutritional status of non-institutionalized individuals in the United States. NHANES employs a cross-sectional study design and employs a stratified, multi-stage probability sampling method for data collection, conducted biennially. The NHANES protocol is approved by the National Center for Health Statistics Research Ethics Review Board, and all participants provide written informed consent. The data for this cross-sectional study were derived from the NHANES database over a continuous period spanning six different time periods (2007–2008, 2009–2010, 2011–2012, 2013–2014, 2015–2016, and 2017–2018). A total of 59,842 participants were initially included in the study. We excluded individuals who did not have CKD (n = 34,870), those with missing dietary data necessary for calculating the DII (n = 9,179), and those with missing serum creatinine data required for estimating the eGFR (n = 13,305). Consequently, a final cohort of 2,488 participants was included in this trial (Fig 1).

### Diagnostic criteria of CKD

We calculated the urine albumin-to-creatinine ratio (ACR) and defined ACR > 30 mg/g as proteinuria. Estimated Glomerular Filtration Rate (eGFR) was calculated using the CKD Epidemiology Collaboration equation, which incorporates gender, age, and serum creatinine levels, and is expressed in ml/min/1.73 m^2^ [14]. An eGFR below 60 ml/min/1.73 m^2^ is typically defined as low eGFR. According to the KDIGO (Kidney Disease: Improving Global Outcomes) guidelines, CKD is defined as the presence of either proteinuria or a low eGFR.

### Exposure variable and outcome variables

In this study, the Dietary Inflammatory Index (DII) is considered as the exposure variable. The 24-hour dietary data obtained through recall interviews were used to calculate the DII score for each individual. The calculation method for DII scores is as previously described [12]. The NHANES dataset contains 26 different food parameters used to compute the DII, including anti-inflammatory food components (alcohol, β-carotene, fiber, folate, magnesium, zinc, selenium, vitamin A, vitamin D, vitamin B-6, vitamin C, vitamin E, monounsaturated fatty acids, niacin, riboflavin, polyunsaturated fatty acids, caffeine, and thiamin) as well as pro-inflammatory food components (cholesterol, carbohydrates, energy, fat, iron, vitamin B-12, protein, and saturated fatty acids). Studies have shown that when calculating DII scores, using fewer than 30 dietary parameters provides the same predictive accuracy as using more than 30 dietary parameters [15,16].

The eGFR and higher CKD stage are considered as the exposure variables.

### Covariates

The criteria for screening covariates in this study were: (1) demographic data (2) variables in the published literature that influence DII and renal function as well as the eGFR index (3) covariates with regression coefficients on the outcome variables with a *P* value <0.10 or covariates that resulted in more than a 10% change in the regression coefficients of the risk factors after introduction of the covariates in the base model (4) other variables selected on the basis of clinical experience.

Therefore, the covariates considered in this study include age, gender, race, education level, marital status, poverty income ratio (PIR), metabolic equivalent (MET) score, diabetes, hypertension, smoking status, alcohol consumption, systolic blood pressure (SBP, mmHg), diastolic blood pressure (DBP, mmHg), body mass index (BMI, kg/m^2^), cotinine level (ng/mL), triglycerides (mmol/L), and cholesterol (mmol/L). The measurement procedures for these variables can be found on the CDC NHANES website at https://www.cdc.gov/nchs/nhanes/.

### Statistical analysis

In accordance with the NHANES database guidelines, statistical analyses were performed on the Dietary Inflammatory Index (DII) and estimated glomerular filtration rate (eGFR). Continuous variables were presented as mean ± standard deviation (SD), while categorical variables were expressed as percentages. We used multiple imputation, based on 5 replications and a chained equation approach method in the R MI procedure, to account for missing data. Initially, we transformed the DII into four quartiles. The chi-squared test was employed to calculate *P* -values for the basic characteristics of analyzed individuals with categorical variables, while the Kruskal-Wallis rank-sum test was used to compute *P* -values for continuous variables. Subsequently, three weighted linear regression models and weighted logistic regression models (Model 1, Model 2, Model 3) were constructed, adjusting for various variables to investigate the association between DII and eGFR and risk of higher CKD stage. Stratified analyses were also conducted based on the fully adjusted model to explore potential stratified associations between DII and eGFR. Additionally, a Generalized Additive Model (GAM) with penalized splines was used to construct smoothed curve fits for the fully adjusted model, treating DII as a continuous variable. The National Center for Health Statistics (NCHS) conducted the survey and received approval from the NCHS Institutional Review Board (IRB). Before data collection and NHANES health examinations, informed consent was obtained from all eligible subjects. (https://www.cdc.gov/nchs/nhanes/irba98.htm).

Furthermore, all authors affirmed that the methods employed in the study adhered to the relevant NHANES Analytic Guidelines. (https://wwwn.cdc.gov/nchs/nhanes/analyticguidelines.aspx#analytic-guidelines).

All statistical analyses were carried out using Empower Stats software and R version 4.2.0. In our study, *P* -values less than 0.05 were considered statistically significant.

## Result

### Baseline characteristics of the participants

Based on the quartiles of the Dietary Inflammatory Index (DII), the baseline characteristics of the study population are presented in Table 1. A total of 2,488 CKD participants were included in our study. The selected participants had an average age of 67.11 years (±15.56), with a predominant representation of non-Hispanic white individuals in the study population. Among the included variables, gender, race, PIR (poverty-to-income ratio), education level, marital status, MET score, serum cotinine, alcohol consumption, CKD stage, being CKD higher stages and eGFR (estimated glomerular filtration rate) levels showed statistically significant differences across the quartiles (*P* -value <0.05).

**Table 1:** Baseline characteristics of the selected participants.

DII	Q1	Q2	Q3	Q4	*P* value
N	621	622	622	622	
Age, mean±SD(years)	(621) 67.39 ± 14.77	(622) 66.78 ± 15.85	(622) 67.13 ± 15.52	(622) 67.16 ± 14.91	0.847
Gender(%)					<0.001
Male	355 (57.17%)	328 (52.73%)	258 (41.48%)	242 (38.91%)	
Female	266 (42.83%)	294 (47.27%)	364 (58.52%)	380 (61.09%)	
Race/ethnicity(%)					0.004
Mexican American	62 (9.98%)	50 (8.04%)	53 (8.52%)	68 (10.93%)	
Other Hispanic	44 (7.09%)	39 (6.27%)	43 (6.91%)	35 (5.63%)	
Non-Hispanic White	328 (52.82%)	327 (52.57%)	297 (47.75%)	269 (43.25%)	
Non-Hispanic Black	148 (23.83%)	173 (27.81%)	196 (31.51%)	219 (35.21%)	
Other Race	39 (6.28%)	33 (5.31%)	33 (5.31%)	31 (4.98%)	
Poverty to income ratio, mean±SD	(552) 2.68 ± 1.56	(577) 2.52 ± 1.53	(566) 2.19 ± 1.42	(560) 2.00 ± 1.35	<0.001
Education(%)					<0.001
Less than high school	149 (23.99%)	170 (27.33%)	221 (35.53%)	257 (41.32%)	
High school	145 (23.35%)	159 (25.56%)	158 (25.40%)	155 (24.92%)	
More than high school	327 (52.66%)	292 (46.95%)	240 (38.59%)	207 (33.28%)	
Not recorded	0 (0.00%)	1 (0.16%)	3 (0.48%)	3 (0.48%)	
Marital status (%)					0.022
Married	333 (53.62%)	305 (49.04%)	269 (43.25%)	278 (44.69%)	
Single	262 (42.19%)	277 (44.53%)	310 (49.84%)	304 (48.87%)	
Living with a partner	16 (2.58%)	21 (3.38%)	21 (3.38%)	20 (3.22%)	
Not recorded	10 (1.61%)	19 (3.05%)	22 (3.54%)	20 (3.22%)	
BMI,mean±SD	(612) 29.83 ± 7.23	(607) 30.47 ± 7.54	(611) 30.57 ± 7.67	(598) 29.86 ± 7.01	0.230
MET	(620) 807.21 ± 2966.62	(621) 477.60 ± 1762.93	(620) 484.97 ± 3289.01	(617) 452.53 ± 1792.03	<0.001
Drinking(%)					0.001
Yes	418 (67.31%)	428 (68.81%)	377 (60.61%)	363 (58.36%)	
No	172 (27.70%)	159 (25.56%)	199 (31.99%)	213 (34.24%)	
Not recorded	31 (4.99%)	35 (5.63%)	46 (7.40%)	46 (7.40%)	
Smoking(%)					0.198
Yes	306 (49.28%)	330 (53.05%)	304 (48.87%)	316 (50.80%)	
No	306 (49.28%)	273 (43.89%)	297 (47.75%)	287 (46.14%)	
Not recorded	9 (1.45%)	19 (3.05%)	21 (3.38%)	19 (3.05%)	
Hypertension history (%)					0.108
Yes	435 (70.05%)	457 (73.47%)	458 (73.63%)	465 (74.76%)	
No	178 (28.66%)	151 (24.28%)	146 (23.47%)	141 (22.67%)	
Not recorded	8 (1.29%)	14 (2.25%)	18 (2.89%)	16 (2.57%)	
Diabetes history (%)					0.087
Yes	196 (31.56%)	223 (35.85%)	235 (37.78%)	236 (37.94%)	
No	399 (64.25%)	386 (62.06%)	368 (59.16%)	372 (59.81%)	
Borderline	26 (4.19%)	13 (2.09%)	18 (2.89%)	14 (2.25%)	
Not recorded	0 (0.00%)	0 (0.00%)	1 (0.16%)	0 (0.00%)	
Cotinine	(619) 32.47 ± 102.79	(622) 41.03 ± 111.93	(620) 52.43 ± 159.21	(622) 57.20 ± 134.76	<0.001
Systolic blood pressure	(577) 134.01 ± 22.64	(577) 135.56 ± 22.87	(561) 134.90 ± 22.96	(567) 136.90 ± 24.48	0.240
Diastolic blood pressure	(572) 67.92 ± 14.00	(568) 67.08 ± 14.03	(553) 66.78 ± 14.56	(559) 66.69 ± 14.47	0.426
Total cholesterol, mean±SD (mmol/L)	(620) 4.77 ± 1.26	(622) 4.76 ± 1.22	(621) 4.82 ± 1.22	(622) 4.78 ± 1.21	0.802
Total triglyceride, mean±SD (g/L)	(620) 1.90 ± 1.31	(622) 1.92 ± 1.33	(621) 1.94 ± 1.70	(622) 1.82 ± 1.17	0.432
CKD Stage (%)					<0.001
Stage 1	74 (11.90%)	60 (9.66%)	52 (8.36%)	49 (7.88%)	
Stage 2	46 (7.40%)	39 (6.28%)	38 (6.11%)	46 (7.40%)	
Stage 3	468 (75.24%)	464 (74.72%)	468 (75.24%)	432 (69.45%)	
Stage 4	27 (4.34%)	35 (5.64%)	41 (6.59%)	58 (9.32%)	
Stage 5	7 (1.13%)	23 (3.70%)	23 (3.70%)	37 (5.95%)	
Higher stage of CKD (%)					<0.001
No	588 (94.53%)	563 (90.66%)	558 (89.71%)	527 (84.73%)	
Yes	34 (5.47%)	58 (9.34%)	64 (10.29%)	95 (15.27%)	
eGFR, mean±SD (ml/min/1.73 m^2^)	(621) 58.26 ± 23.64	(622) 54.42 ± 24.95	(622) 53.32 ± 24.75	(622) 51.31 ± 25.40	<0.001

Note: Q1–Q4: Grouped by quartile according to DII.

Mean ± SD for continuous variables: *P* value was calculated by Kruskal-Wallis rank-sum test % for categorical variables: *P* value was calculated by chi-square test.

### Association between DII and higher CKD stage

Weighted multiple logistic regression was used to analyze the correlation between DII and the risk of higher stages of CKD, and the results are shown in Table 2. The association between DII and the risk of higher-stage CKD was positive in all multivariate logistic regression models, regardless of whether confounding variables were adjusted for (model 1: OR = 1.31, 95% CI: 1.20–1.43; Model 2: OR = 1.27, 95% CI: 1.17–1.39; Model 3: OR = 1.26, 95% CI: 1.14–1.40). After adjusting for the potential confounding variable (Model 3), the risk of higher CKD stage increased by 26% for every 1 unit increase in DII. The trend in the DII quartiles remained statistically significant, with the risk of higher stages of CKD gradually increasing from the second, third, and fourth DII quartiles compared to the lowest quartile (*P* for trend < 0.005).

**Table 2:** Multivariate weighted logistic regression model reveals the associations between DII and higher CKD stage.

	Model 1	Model 2	Model 3
	**OR (95% CI) *P* value**	**OR (95% CI) *P* value**	**OR (95% CI) *P* value**
DII	1.31 (1.20, 1.43) <0.0001	1.28 (1.17, 1.39) <0.0001	1.26 (1.14, 1.40) <0.0001
DII (Quartile)			
Q1 (-4.86–0.31)	1.0	1.0	1.0
Q2 (0.32–1.94)	1.78 (1.15, 2.76) 0.0099	1.76 (1.13, 2.73) 0.0122	1.46 (0.88, 2.40) 0.1402
Q3 (1.95–2.99)	1.98 (1.29, 3.05) 0.0019	1.84 (1.19, 2.85) 0.0059	1.63 (0.99, 2.69) 0.0548
Q4 (3.00–5.05)	3.12 (2.07, 4.69) <0.0001	2.82 (1.86, 4.26) <0.0001	2.29 (1.42, 3.71) 0.0007
*P* for trend	<0.0001	<0.0001	0.0007

Model 1: No covariates were adjusted.

Model 2: Age, gender, and race were adjusted.

Model 3: Fully adjusted model.

### Association between DII and eGFR

A weighted multiple regression analysis was conducted to examine the association between DII and eGFR, and the results are presented in Table 3. In all three models, an increase in DII was found to be significantly associated with a decrease in eGFR (*P* < 0.0001). In the fully adjusted model, the result was as follows: β = -1.29 (-1.75, -0.83). The trend in DII quartiles remained statistically significant, with eGFR levels progressively decreasing from the second, third, and fourth DII quartiles compared to the lowest quartile (*P* for trend < 0.0001). As showed in S1 and S2 Tables in S1 File and S1 Fig in S1 File, we conducted sensitivity analysis using the data after multiple imputation. The results indicated that the imputed data results were nearly identical to the original data results.

**Table 3:** Multivariate weighted regression model analysis reveals the associations between DII and eGFR.

	Model 1	Model 2	Model 3
	β (95% CI) *P* value	β (95% CI) *P* value	β (95% CI) *P* value
DII	-1.57 (-2.09, -1.05) <0.0001	-1.31 (-1.73, -0.88) <0.0001	-1.29 (-1.75, -0.83) <0.0001
DII (Quartile)			
Q1 (-4.86–0.31)	Reference	Reference	Reference
Q2 (0.32–1.94)	-1.97 (-4.52, 0.58) 0.1301	-2.70 (-4.74, -0.65) 0.0099	-2.76 (-4.91, -0.60) 0.0123
Q3 (1.95–2.99)	-5.70 (-8.34, -3.06) <0.0001	-4.04 (-6.17, -1.90) 0.0002	-3.39 (-5.67, -1.11) 0.0036
Q4 (3.00–5.05)	-7.03 (-9.69, -4.37) <0.0001	-5.95 (-8.11, -3.79) <0.0001	-5.29 (-7.62, -2.96) <0.0001
*P* for trend	<0.0001	<0.0001	<0.0001

Model 1: No covariates were adjusted.

Model 2: Age, gender, and race were adjusted.

Model 3: Fully adjusted model.

### Stratified associations between DII and eGFR

We conducted a stratified analysis to examine the stability of the regression analysis results between DII and eGFR in different subgroups, and the results are presented in Table 4. In the subgroup analysis, DII was found to be negatively associated with eGFR in most subgroups, and these associations were statistically significant. The exceptions were age≤30, Other Hispanic ethnicity, living with a partner, low PIR, borderline diabetes history, and high cholesterol. Furthermore, it appears that variables such as marital status, hypertension history, and diabetes history may have interaction effects with eGFR (*P* for interaction < 0.05). The fitting plot of the subgroup smooth curve constructed based on the fully adjusted model is shown in S2 Fig in S1 File.

**Table 4:** The stratified analysis of the association between DII and eGFR.

	eGFR	
Subgroups	β (95% CI) *P* value	*P* for interaction
**Age**		0.0541
< = 30	1.30 (-2.02, 4.61) 0.4433	
>30, < = 60	-1.90 (-2.80, -1.00) <0.0001	
>60	-0.90 (-1.42, -0.38) 0.0007	
**Gender**		0.903
Male	-1.40 (-2.08, -0.72) <0.0001	
Female	-1.34 (-1.95, -0.74) <0.0001	
**Race**		0.4358
Mexican American	-2.68 (-4.89, -0.46) 0.0181	
Other Hispanic	-0.70 (-3.58, 2.19) 0.6353	
Non-Hispanic White	-1.03 (-1.56, -0.51) 0.0001	
Non-Hispanic Black	-1.57 (-2.75, -0.39) 0.0091	
Other Race	-2.36 (-4.65, -0.06) 0.0442	
**Education**		0.3977
Less than high school	-1.86 (-2.87, -0.84) 0.0003	
High school	-1.35 (-2.22, -0.48) 0.0023	
More than high school	-1.05 (-1.67, -0.43) 0.0009	
**Marital status**		0.0048
Married	-1.02 (-1.63, -0.41) 0.0011	
Single	-1.90 (-2.57, -1.22) <0.0001	
Living with a partner	3.10 (0.06, 6.14) 0.0457	
**Poverty to income ratio**		0.0048
Low	-0.70 (-1.64, 0.23) 0.1398	
Middle	-2.04 (-2.84, -1.24) <0.0001	
High	-1.07 (-1.76, -0.39) 0.0021	
**BMI**		0.7526
< = 25	-1.38 (-2.28, -0.48) 0.0026	
25–30	-1.10 (-1.88, -0.31) 0.0063	
>30	-1.50 (-2.21, -0.78) <0.0001	
**MET**		0.0884
0	-2.05 (-2.83, -1.27) <0.0001	
0–500	-0.88 (-1.68, -0.08) 0.0316	
>500	-1.13 (-1.89, -0.37) 0.0037	
Smoking		0.5824
Yes	-1.14 (-1.78, -0.50) 0.0005	
No	-1.35 (-1.98, -0.72) <0.0001	
**Drinking**		0.3421
Yes	-1.07 (-1.62, -0.52) 0.0001	
No	-1.81 (-2.67, -0.95) <0.0001	
**Diabetes history**		0.0263
Yes	-2.31 (-3.16, -1.46) <0.0001	
No	-1.08 (-1.62, -0.54) <0.0001	
Borderline	1.16 (-3.32, 5.64) 0.6114	
**Hypertension history**		0.0125
Yes	-1.05 (-1.60, -0.51) 0.0001	
No	-1.42 (-2.20, -0.63) 0.0004	
**Cotinine**		0.8303
Low	-1.41 (-2.12, -0.71) <0.0001	
Middle	-1.08 (-1.93, -0.24) 0.0119	
High	-1.20 (-2.02, -0.39) 0.0038	
**Systolic blood pressure**		0.2565
Low	-1.10 (-1.84, -0.36) 0.0038	
Middle	-0.79 (-1.57, -0.01) 0.0466	
High	-1.72 (-2.55, -0.89) <0.0001	
**Diastolic blood pressure**		0.0664
Low	-1.25 (-2.04, -0.47) 0.0018	
Middle	-2.03 (-2.84, -1.22) <0.0001	
High	-0.77 (-1.49, -0.04) 0.0385	
**Total cholesterol**		0.1248
Low	-1.25 (-2.05, -0.45) 0.0022	
Middle	-1.74 (-2.54, -0.94) <0.0001	
High	-0.63 (-1.37, 0.11) 0.0931	
**Total triglyceride**		0.2913
Low	-1.33 (-2.10, -0.55) 0.0008	
Middle	-1.68 (-2.46, -0.89) <0.0001	
High	-0.82 (-1.59, -0.05) 0.0375	

Subgroup analysis was constructed based on a fully adjusted model. In each case, the model was not adjusted for the stratification variable itself.

### Non-linear relationships

**Fig 2:**
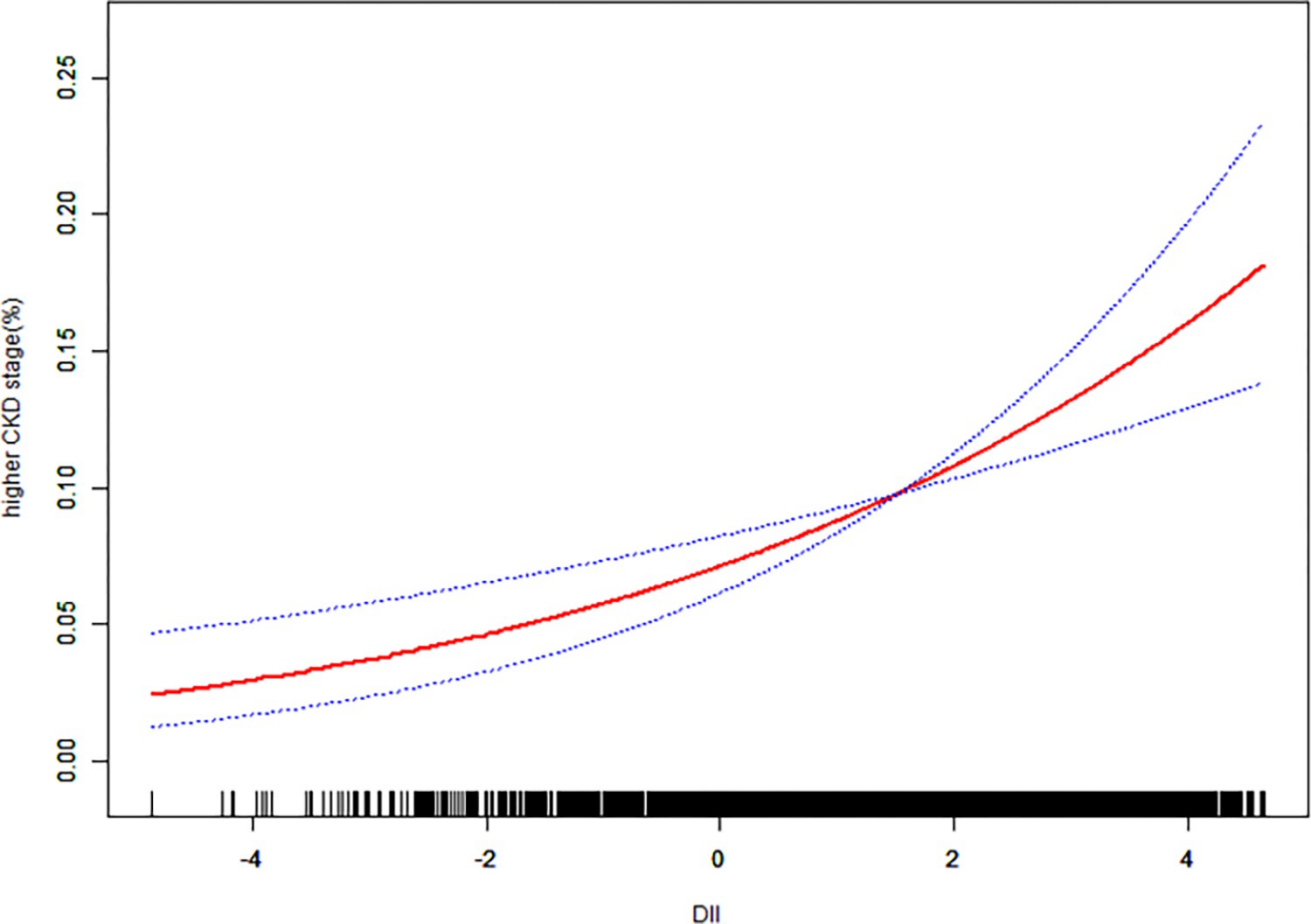
Based on the fully adjusted model, the relationship between DII and higher CKD stage. The solid red line represents the smooth fitting curve between variables, and the blue band represents the 95% CI of the fitting.

**Fig 3:**
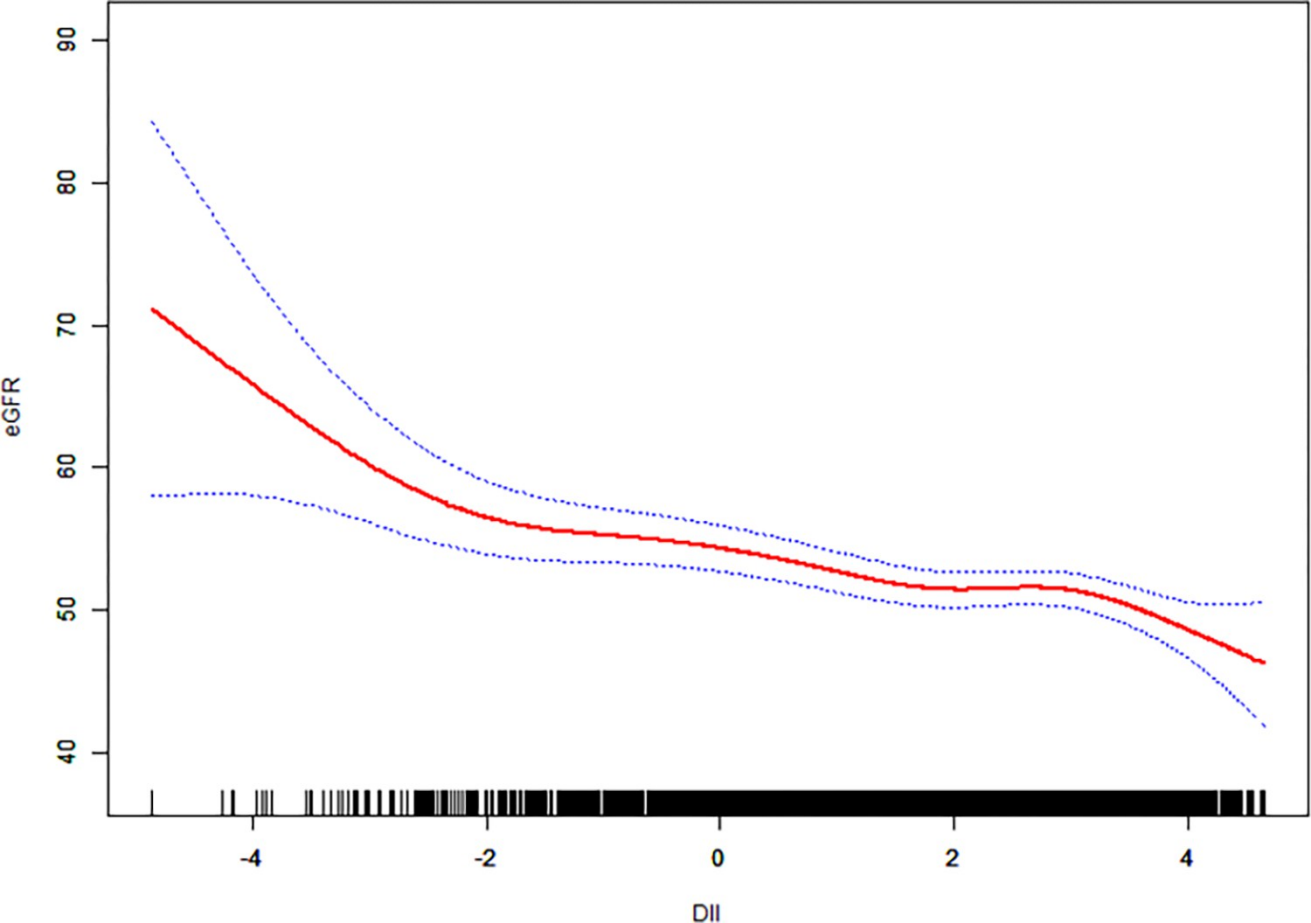
Based on the fully adjusted model, the relationship between DII and eGFR. The solid red line represents the smooth fitting curve between variables, and the blue band represents the 95% CI of the fitting.

To ensure the reliability of the regression analysis results, we employed a Generalized Additive Model (GAM) and smoothed curve fitting to examine whether there was a linear or non-linear relationship between DII and eGFR and between DII and the risk of higher CKD stage. In our study, based on the fully adjusted model (Model 3), we constructed smoothed curve fits to observe potential correlations. The results are shown in Figs 2 and 3. We observed a nonlinear negative correlation between DII and eGFR, and a nonlinear positive correlation between DII and the risk of higher CKD stage. As DII increases, eGFR tends to decrease, and the risk of higher CKD stage increases.

## Discussion

To the best of our knowledge, this study is the first to investigate the relationship between DII and the risk of higher CKD stage and the kidney function indicator eGFR in American CKD patients. We examined the correlation between DII scores and the risk of higher CKD stage and eGFR in 2,488 CKD patients who participated in the US NHANES survey from 2007 to 2018. In the fully adjusted model, we found that an increase in DII score of one unit was associated with a 26% increase in the risk of higher CKD stage and a 1.49 decrease in eGFR levels. Our subgroup analysis and smoothed curve fitting results further validate the stability and reliability of our regression analysis findings.

This finding aligns with research results from published studies. A cross-sectional study involving Iranian adults showed that individuals with higher DII scores had lower eGFR levels [10]. Another cross-sectional study focusing on the Iranian CKD population revealed an increased risk of advanced CKD stages in the highest DII quartile group [17]. An observational study involving American adults indicated that as the E-DII increased, the average eGFR significantly decreased [18]. A cohort study involving older adults found that a pro-inflammatory diet assessed by the Adapted Dietary Inflammatory Index (ADII) was associated with systemic inflammation and declining kidney function in older individuals, with CRP playing a significant mediating role, suggesting that diet can induce kidney damage through inflammation [19]. Moreover, a study examining the relationship between diet and proteinuria in a multi-ethnic population demonstrated that an anti-inflammatory dietary pattern rich in whole grains, fruits(low in potassium, such as papaya, watermelon, apples and apricot plum), and low-fat dairy products was associated with reduced urinary albumin-to-creatinine ratio (ACR) [20]. Similarly, another study showed that a pro-inflammatory dietary pattern with higher animal fat intake was associated with an increased risk of microalbuminuria, while lower sodium and higher β-carotene intake (anti-inflammatory) may reduce the risk of eGFR decline [21]. Pro-inflammatory diets not only harm kidney function in chronic kidney disease patients but also elevate the risk of kidney cancer. A cohort study conducted in Iowa showed that higher DII scores, indicative of a pro-inflammatory dietary pattern, were associated with an increased risk of kidney cancer [22].

Prior research has indicated that inflammation plays an indispensable role in the progression of CKD. Inflammation can induce the release of cytokines, produce adhesion molecules, and increase their activity, promoting the progression of CKD [23]. Furthermore, one potential mechanism of the association between DII and kidney diseases is that a pro-inflammatory diet can induce chronic inflammation, leading to the upregulation of various pro-inflammatory factors, such as CRP [24]. CRP, one of the inflammation markers used in calculating DII, is an independent predictor of cardiovascular disease in end-stage kidney disease patients [25]. Some studies have shown that an increase in CRP is associated with a decrease in creatinine clearance in pre-dialysis chronic kidney failure patients [26] and may serve as a risk marker for kidney function impairment [27]. Moreover, CRP is a good predictor of malnutrition and the overall and cardiovascular disease mortality rates in CKD patients [28,29]. DII, as a comprehensive dietary index, has the capability to assess the overall inflammatory potential of the diet, [30] potentially reducing renal impairment and mortality rates in CKD patients.

The KDOQI guidelines recommend that patients with CKD reduce their daily intake of potassium, sodium, phosphorus and protein [31]. Protein is a pro-inflammatory nutrient in the DII calculation [12]. Dietary recommendations for patients with CKD should be < 100 mg/day for phosphorus and 2400 mg/day for potassium [31]. It is important to note that although potassium and phosphorus are not included in the DII calculation, potassium and phosphorus are likely to have an anti-inflammatory aspect, as nutrients that are readily present with potassium, such as vitamin C and flavonols, are anti-inflammatory, and nutrients that are readily present with plant phosphorus, such as fibre and flavonoids, are anti-inflammatory [12]. Therefore, it is best for people with CKD to pay particular attention to the amount of potassium and phosphorus in their daily diet, and to avoid eating fruits, vegetables, nuts and other foods that are high in potassium and phosphorus.

This study has some limitations. Firstly, like other cross-sectional studies, we cannot establish a causal relationship between DII and eGFR, as our study design is observational. Secondly, although we adjusted for some confounding factors, there may be other potential confounding factors that were not considered. Additionally, our survey was based on NHANES data, which is specific to the U.S. population, and further research is needed to confirm the relationship between DII and eGFR in a more diverse population. Moreover, due to data limitations in NHANES, it is not possible to tell from the data provided by the database whether the participants have adhered to the renal diet. However, our strength lies in the use of DII scores to assess the inflammatory potential of diets, which relies on research findings from scientific publications rather than population averages or intake recommendations. Additionally, the use of a nationally representative high-quality sample adds to the representativeness of our results. Furthermore, we adjusted for many confounding factors and conducted multivariate regression, subgroup analysis, and smoothed curve fitting, making our findings more reliable.

## Conclusion

Our study results indicate that an increase in DII is associated with an increased risk of higher CKD stage and a decrease in eGFR in all three models. In the fully adjusted model, the risk of higher CKD stage increased by 26% and the eGFR decreased by 1.29 ml/min/1.73 m^2^ for each unit increase in DII. This finding suggests that in patients with CKD in the US, improved diet and lower DII values may help slow the decline in eGFR and delay the progression of CKD.

## Supporting information

S1 File (DOCX)
