# Supplementary material for: Association of Dietary Inflammatory Index with CKD progression and estimated glomerular filtration rate in the American CKD population: A cross-sectional study
Source: PLoS One. 2024 Feb 22;19(2):e0297916. doi: 10.1371/journal.pone.0297916 (PMC10883550; doi:10.1371/journal.pone.0297916)

**S1 Table. Comparison of Multiple Imputation Data and Original Data Multivariate Regression Analysis Results**

| Model | MI.ITER= 0 | MI.ITER= 1 | MI.ITER= 2 | MI.ITER= 3 | MI.ITER= 4 | MI.ITER= 5 |
| --- | --- | --- | --- | --- | --- | --- |
| Non-adjusted | 1.31 (1.20, 1.43) <0.0001 | 1.31 (1.20, 1.43) <0.0001 | 1.31 (1.20, 1.43) <0.0001 | 1.31 (1.20, 1.43) <0.0001 | 1.31 (1.20, 1.43) <0.0001 | 1.31 (1.20, 1.43) <0.0001 |
| Adjust I | 1.28 (1.17, 1.39) <0.0001 | 1.28 (1.17, 1.39) <0.0001 | 1.28 (1.17, 1.39) <0.0001 | 1.28 (1.17, 1.39) <0.0001 | 1.28 (1.17, 1.39) <0.0001 | 1.28 (1.17, 1.39) <0.0001 |
| Adjust II | 1.26 (1.14, 1.40) <0.0001 | 1.29 (1.17, 1.41) <0.0001 | 1.29 (1.17, 1.41) <0.0001 | 1.29 (1.18, 1.41) <0.0001 | 1.29 (1.17, 1.41) <0.0001 | 1.28 (1.17, 1.41) <0.0001 |

MI.ITER=0 represents the original data; MI.ITER=1-5 represents imputed data.

| Model | MI.ITER= 0 | MI.ITER= 1 | MI.ITER= 2 | MI.ITER= 3 | MI.ITER= 4 | MI.ITER= 5 |
| --- | --- | --- | --- | --- | --- | --- |
| Non-adjusted | -1.57 (-2.09, -1.05) <0.0001 | -1.57 (-2.09, -1.05) <0.0001 | -1.57 (-2.09, -1.05) <0.0001 | -1.57 (-2.09, -1.05) <0.0001 | -1.57 (-2.09, -1.05) <0.0001 | -1.57 (-2.09, -1.05) <0.0001 |
| Adjust I | -1.31 (-1.73, -0.88) <0.0001 | -1.31 (-1.73, -0.88) <0.0001 | -1.31 (-1.73, -0.88) <0.0001 | -1.31 (-1.73, -0.88) <0.0001 | -1.31 (-1.73, -0.88) <0.0001 | -1.31 (-1.73, -0.88) <0.0001 |
| Adjust II | -1.29 (-1.75, -0.83) <0.0001 | -1.44 (-1.85, -1.02) <0.0001 | -1.45 (-1.86, -1.03) <0.0001 | -1.44 (-1.85, -1.02) <0.0001 | -1.45 (-1.86, -1.04) <0.0001 | -1.44 (-1.85, -1.03) <0.0001 |

MI.ITER=0 represents the original data; MI.ITER=1-5 represents imputed data.

**S2 Table. The pooled results of multiple imputation.**

| Outcomes | beta 1 | se 1 | beta 2 | se 2 | beta 3 | se 3 | beta 4 | se 4 | beta 5 | se 5 |
| --- | --- | --- | --- | --- | --- | --- | --- | --- | --- | --- |
| risk of higher CKD stage | 1.29 | 0.061224 | 1.29 | 0.061224 | 1.29 | 0.058673 | 1.29 | 0.061224 | 1.28 | 0.061224 |
| eGFR | -1.44 | 0.211735 | -1.45 | 0.211735 | -1.44 | 0.211735 | -1.45 | 0.209184 | -1.44 | 0.209184 |

| Outcomes | Coefficients | Se | T | P value |
| --- | --- | --- | --- | --- |
| risk of higher CKD stage | 1.288000 | 0.060887 | 21.153830 | 0.000000 |
| eGFR | -1.444000 | 0.210789 | -6.850447 | 0.000000 |


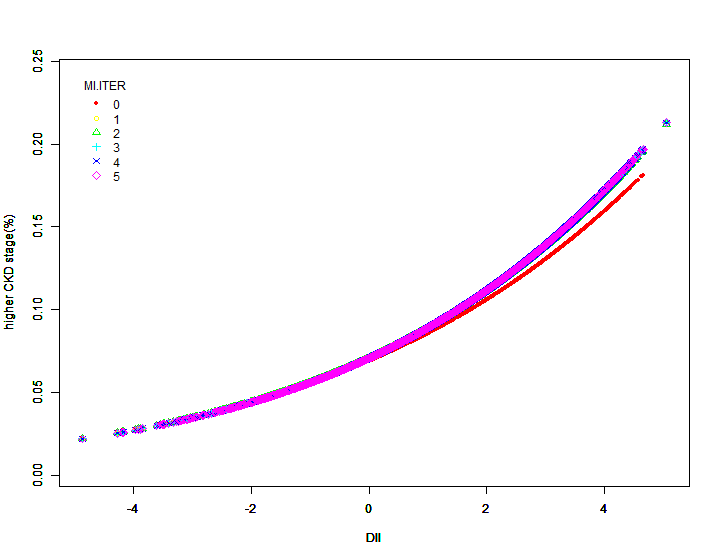

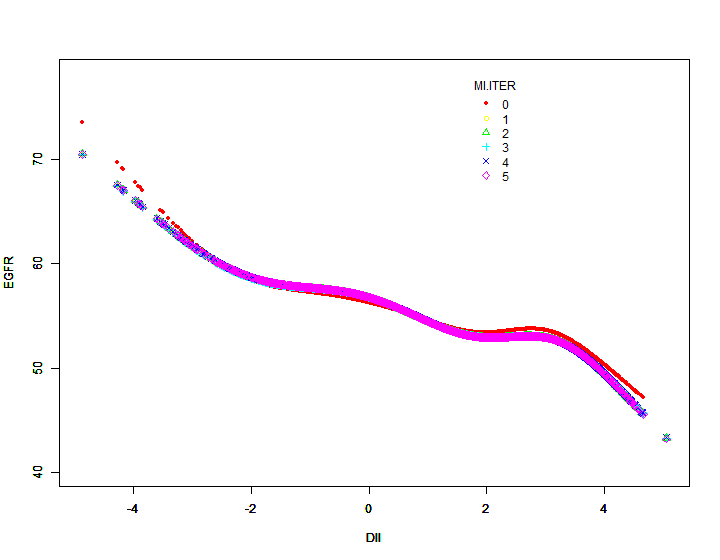


**S1 Fig. Smoothed curve fitting plot for data before and after multiple imputation.** MI.ITER=0 represents the original data; MI.ITER=1-5 represents imputed data.

**S2 Fig. Subgroup smooth curve fitting plot constructed based on fully adjusted model.**


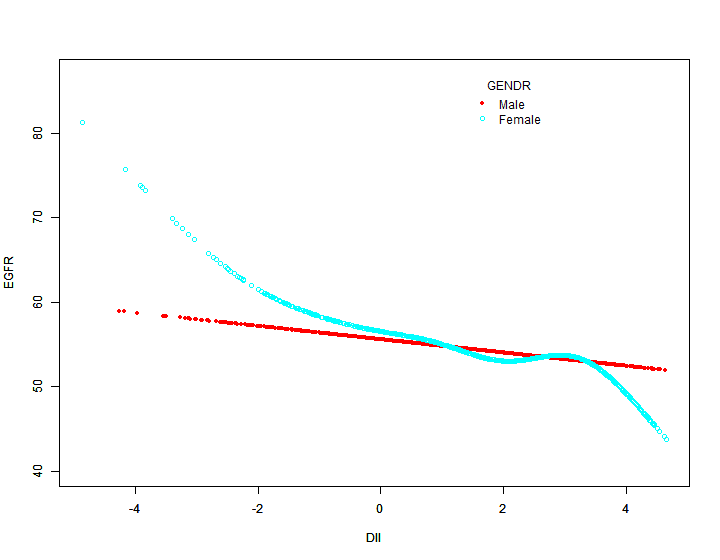

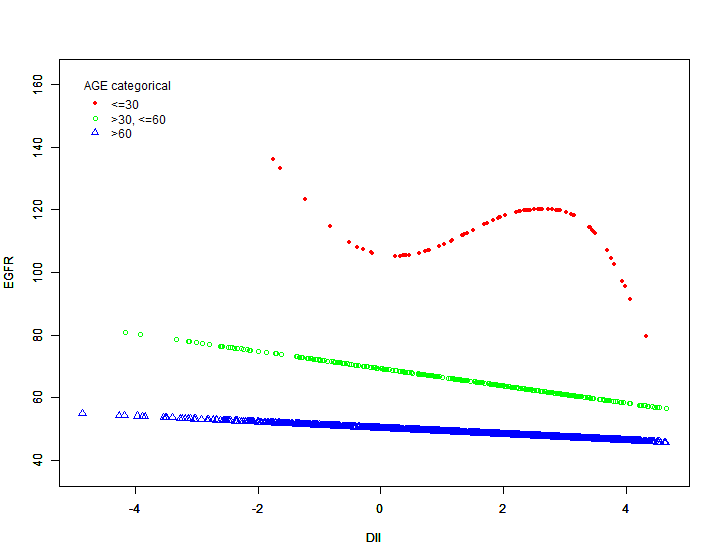

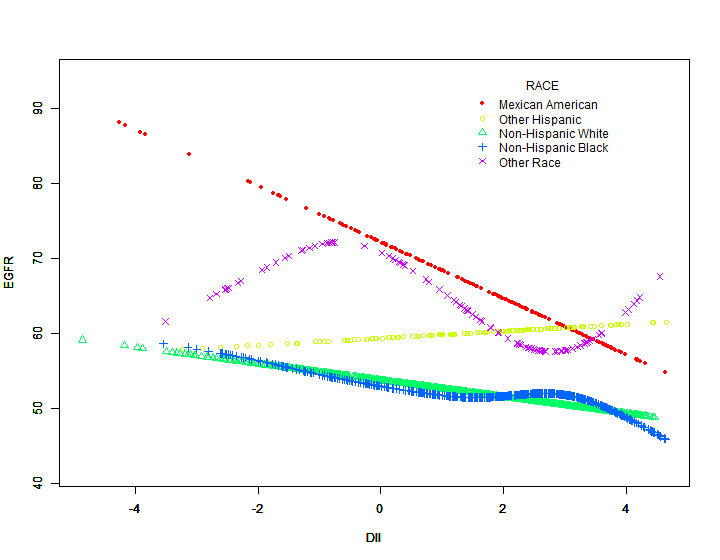

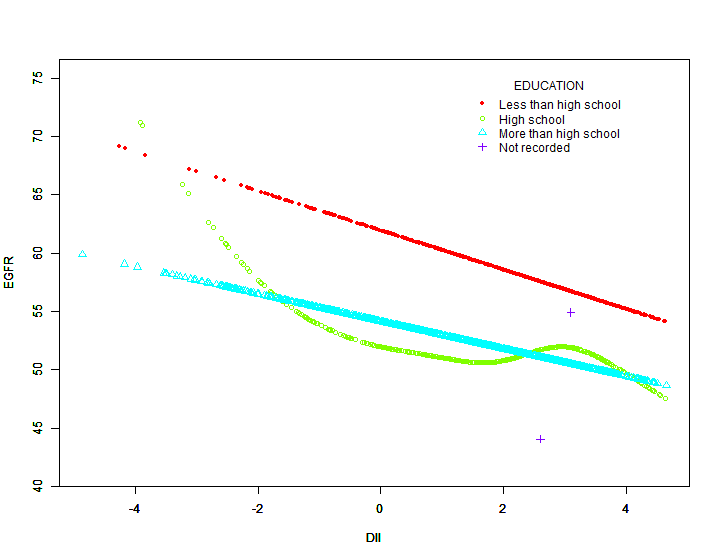

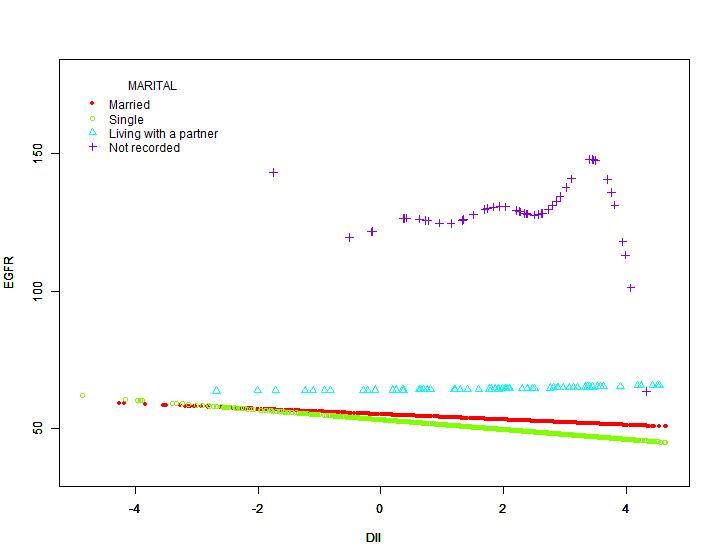

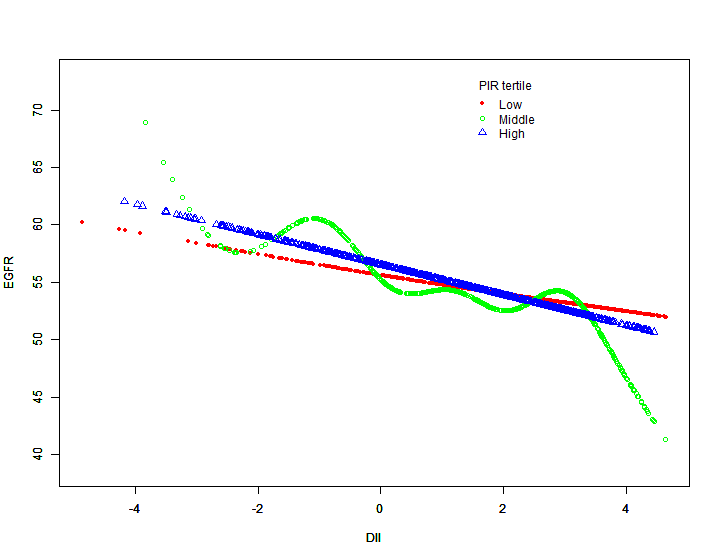

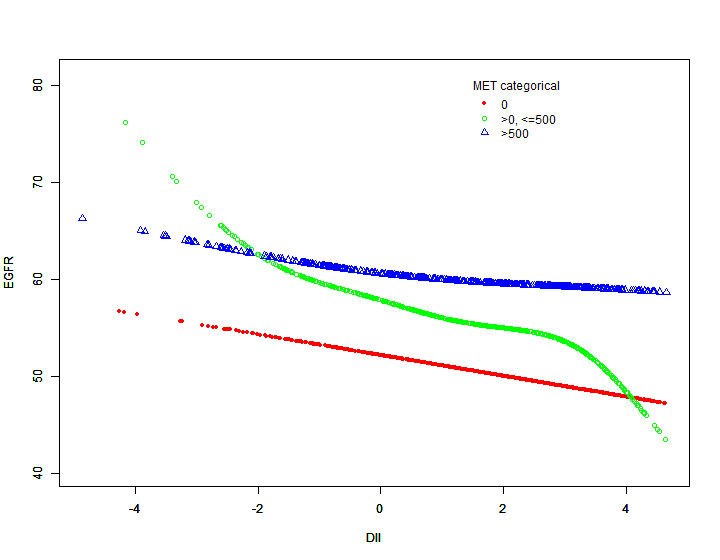

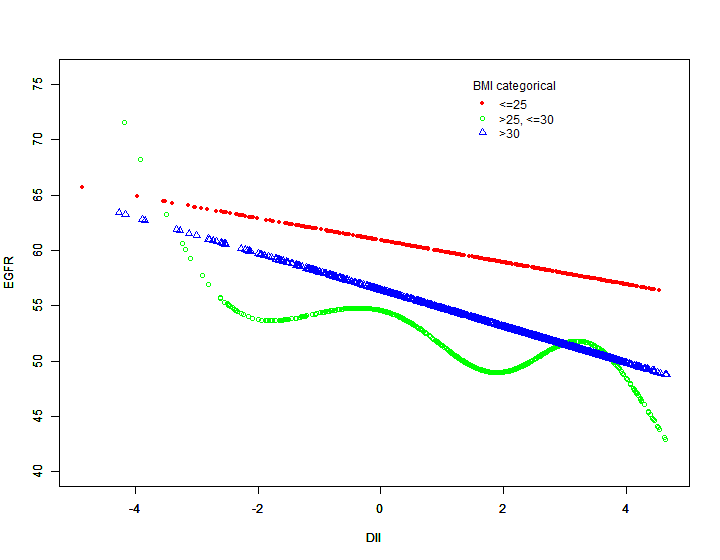

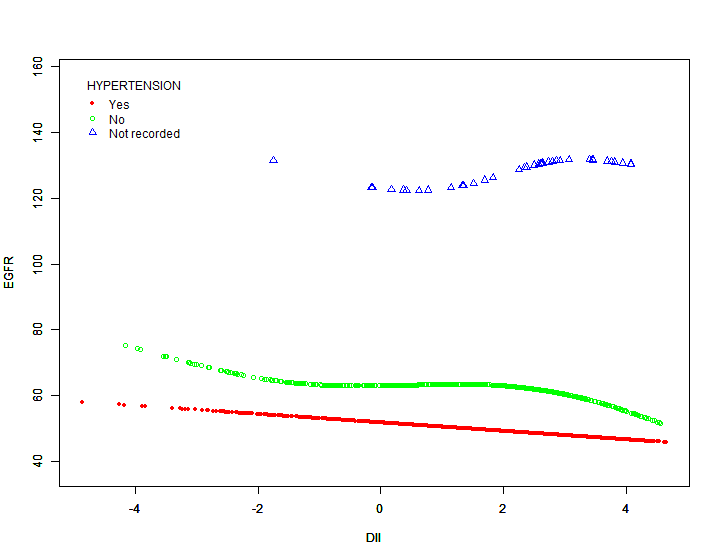

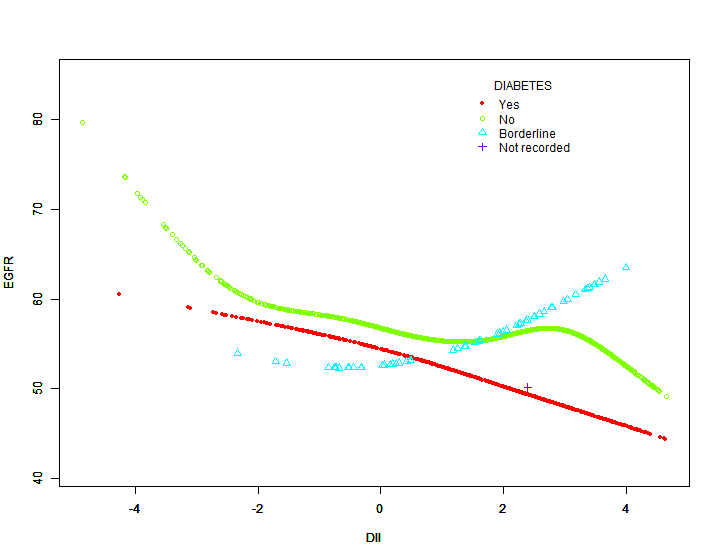

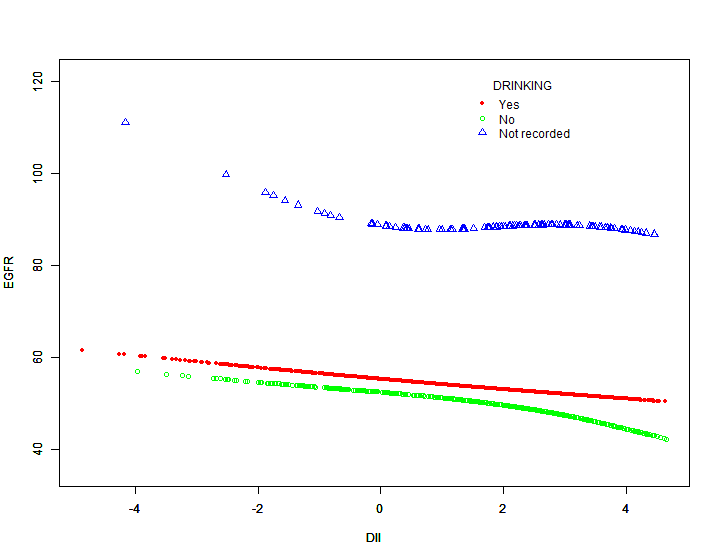

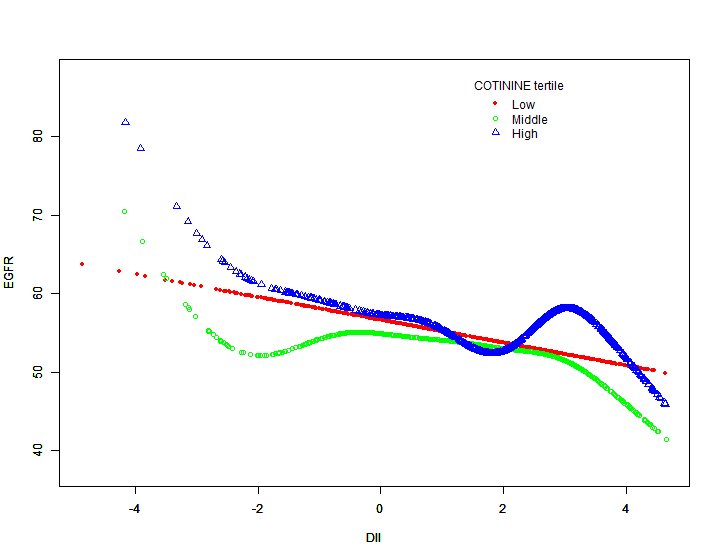

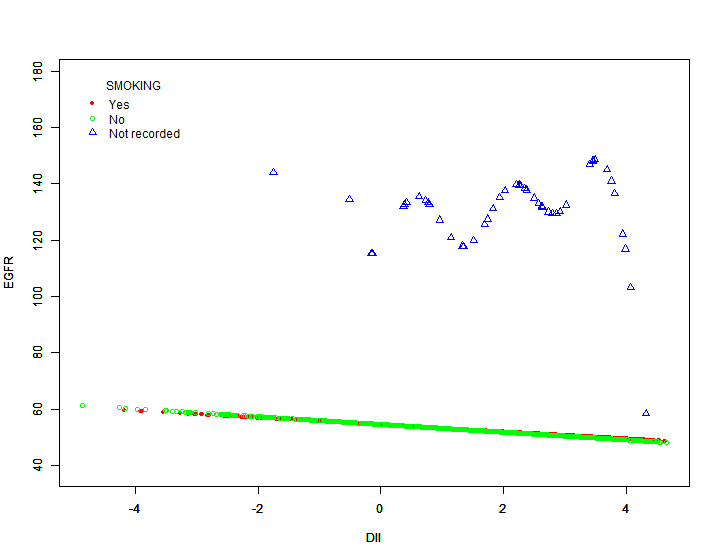

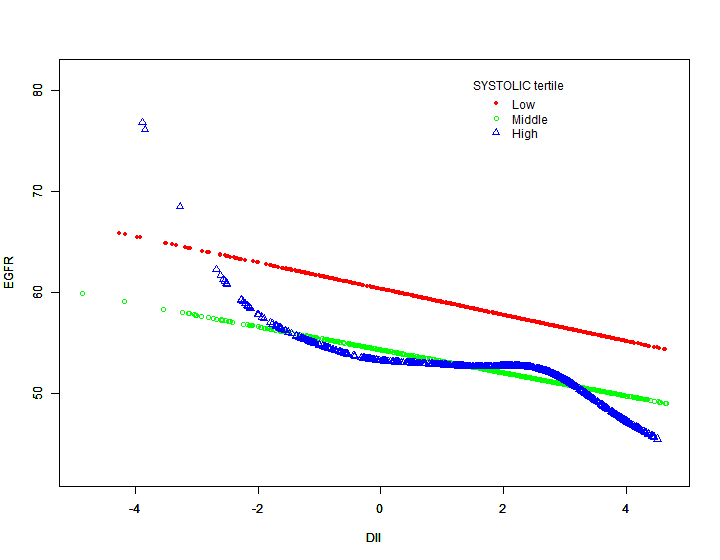

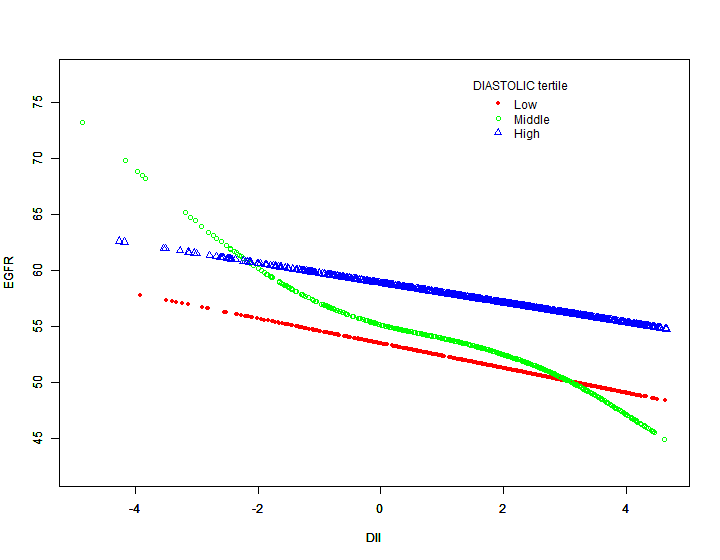

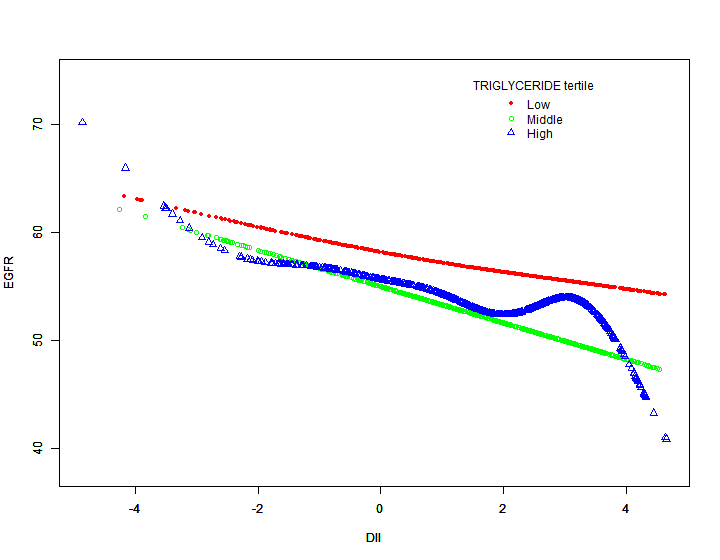

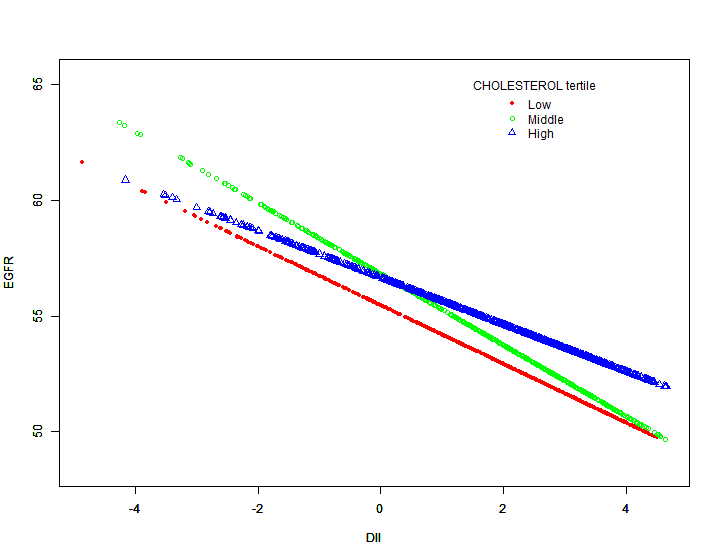

Supplement: S1 File — (DOCX) [file pone.0297916.s001.docx]
